# Evaluation of *Legionella pneumophila* confirmation methods following IDEXX Legiolert analysis of groundwater

**DOI:** 10.1128/spectrum.01228-26

**Published:** 2026-06-30

**Authors:** Christina M. Morrison, Phillip Wang, Gabriela Portillo-Chamul, John Monahan, Kai Chung, Daniel Gerrity

**Affiliations:** 1 Southern Nevada Water Authority 235127, Las Vegas, Nevada, USA; 2 Department of Civil and Environmental Engineering and Construction, University of Nevada 14722, Las Vegas, Nevada, USA; University of Texas Medical Branch at Galveston, Galveston, Texas, USA

**Keywords:** *Legionella*, *L. pneumophila*, Legiolert, MALDI, latex agglutination, qPCR, groundwater

## Abstract

The IDEXX Legiolert test has been implemented for the quantification of *Legionella pneumophila* in a variety of potable and non-potable water matrices. For example, through coordination with the state regulatory agency, the Southern Nevada Water Authority (SNWA) has implemented treatment strategies and corresponding *L. pneumophila* monitoring (via Legiolert) to ensure groundwater quality and public health protection. Because of its specificity to *L. pneumophila*, confirmation of a positive Legiolert test is often assumed to be unnecessary, although high false positive rates have previously been reported for Legiolert testing of groundwater systems. This study evaluated four confirmation methodologies on liquid media harvested from Legiolert-positive trays: latex agglutination, MALDI-MS, rapid qPCR for the *mip* and *srkA* genes, and BCYE plating (±L-cysteine). With the exception of BCYE, these confirmation methods provide results in <24 h, allowing for rapid regulatory and/or public health response. Of 108 presumptive positive analyses of groundwater samples collected in Southern Nevada, only two were ultimately determined to be false positive Legiolert results, although one prompted the shutdown of the corresponding groundwater well. qPCR of the *mip* gene had the highest *L. pneumophila* confirmation rate (96%), followed by MALDI-MS (92%), latex agglutination (88%), and BCYE (±L-cysteine) (88%). Although Legiolert alone proved to be effective and relatively accurate in detecting *L. pneumophila*, confirmation testing yielded valuable supporting information, including serogroup determination and false positive identification (*Stenotrophomonas maltophilia*). Collectively, these data highlight the benefits and drawbacks of various *L. pneumophila* confirmation methodologies for reporting and decision-making by SNWA and other drinking water utilities.

**IMPORTANCE:**

In the United States, *Legionella pneumophila* is the leading cause of drinking water-associated illnesses, hospitalization, and deaths. It is the causative agent of Legionnaires’ disease and the less severe Pontiac Fever. As awareness of *L. pneumophila* risks increases and monitoring plans are implemented, it is imperative that laboratory analysts, practitioners, and decision-makers understand the limitations of available methods and the value of confirmation in increasing data confidence and informing appropriate actions. Groundwater is commonly used as a drinking water source for public and private systems and generally has less stringent treatment requirements than other sources. Studies have described groundwater as an environmental reservoir for *L. pneumophila*, but there have also been reports of high false positive rates when monitoring groundwater, particularly when using IDEXX Legiolert. Therefore, the results from this study provide critical knowledge to those monitoring *L. pneumophila* and using the data for regulatory compliance and/or operational decision-making.

## INTRODUCTION

*Legionella pneumophila* is an intracellular opportunistic pathogen that is the causative agent of Legionnaires’ disease (LD) (1). In the United States and Europe, *L. pneumophila* is the leading cause of drinking water-associated illnesses, hospitalizations, and deaths (2, 3). It is found ubiquitously in both natural and engineered aquatic environments (4), often proliferating within premise plumbing systems where its natural hosts, free living amoeba (FLA), reside in biofilms (5–7). Unlike many waterborne pathogens, *L. pneumophila* is not associated with person-to-person transmission via the fecal-oral route. Rather, *L. pneumophila* is mainly acquired through environmental exposure to aerosols. Outbreaks of LD often occur in large buildings, such as hotels and hospitals, with complicated premise plumbing systems and ample exposure opportunities (e.g., showers) (8). Additionally, outbreaks have been associated with cooling towers capable of spreading aerosolized *L. pneumophila* over large distances (9–11).

Currently, responsibility for *L. pneumophila* control largely falls upon building managers through requirements for developing and implementing water management plans (12). In the United States, there are no *Legionella* monitoring requirements mandated by the Environmental Protection Agency (EPA), and it is only indirectly regulated through the Surface Water Treatment Rule (SWTR), although it is simply assumed to be controlled as long as corresponding virus and protozoa requirements are satisfied (13). The Ground Water Rule (GWR) also has no specific stipulations for *Legionella,* although guidance has been developed in some states (e.g., Nevada) (14). Current water disinfection technologies are effective at *L. pneumophila* inactivation and control within the distribution system (15–17). However, there are many small water systems and residences that rely on groundwater with minimal to no treatment, such as those on private wells. Historically, groundwater has not been considered a significant source of *L. pneumophila* (4, 18), but there are several recent studies demonstrating its presence within public groundwater systems and premise plumbing systems supplied by private groundwater wells (14, 19, 20). In fact, Atkinson et al. (14) observed groundwater concentrations exceeding the U.S. Centers for Disease Control and Prevention (CDC) suggested action level of 100 colony-forming units (CFU)/100 mL (21), particularly after long periods of stagnation.

In Southern Nevada, up to approximately 60 groundwater production wells typically operate from late spring to early fall to alleviate peak demand by supplementing the primary surface water supply from Lake Mead. These wells remain stagnant between fall and spring, necessitating an annual cleanup (i.e., flushing) at the start of well season. Upon delivery to customers, the wells are dosed with sodium hypochlorite to achieve an initial free chlorine residual of 1 mg/L, and distribution system residuals are maintained at >0.5 mg/L. Historically, ~40% of the wells have fed storage reservoirs with average free chlorine Cts of 39–164 mg-min/L, while ~60% of the wells have operated in a direct-to-distribution configuration. Proactive monitoring by the Southern Nevada Water Authority (SNWA) noted frequent detection of *L. pneumophila* in untreated groundwater, particularly prior to flushing, and in direct-to-distribution wells (14), which prompted their shutdown in 2018 until updated *L. pneumophila* guidance could be developed. Developed in collaboration with the Nevada Division of Environmental Protection (NDEP), the updated guidance (22) allows for operation of groundwater wells feeding storage reservoirs, select direct-to-distribution wells with historically low *L. pneumophila* concentrations (<5 MPN/100 mL), and other direct-to-distribution wells with enhanced disinfection, specifically free chlorine coupled with low-pressure UV or UV LED (23). In addition, routine monitoring must demonstrate that *L. pneumophila* concentrations remain <100 MPN/100 mL at all times, with a treatment-based goal of ≤5 MPN/100 mL.

This routine monitoring for *Legionella* spp. or *L. pneumophila* can be accomplished by several methodologies, each with its own weaknesses and strengths. Cultivation on buffered charcoal yeast extract (BCYE) agar via membrane filtration or direct plating (ISO method 11731:2017) can provide concentration data on viable *Legionella* spp., assuming they have not entered a viable but nonculturable (VBNC) state. However, not all species of *Legionella* are able to be cultivated, limiting the ability to fully capture the range of *Legionella* species in a given water sample (ISO 11731). Membrane filtration and plating on BCYE agar can be time- and labor-intensive, require up to 10 days of incubation prior to reaching an initial result, and are susceptible to non-target growth in applications focused on *L. pneumophila*. Thus, further testing is required when *L. pneumophila* confirmation is warranted. Another cultivation-based technique that is specific to *L. pneumophila*, captures only viable organisms, and is amenable to high-throughput monitoring, thus making it attractive to drinking water utilities (24), is Legiolert (IDEXX, Westbrook, ME). The 7-day Legiolert test utilizes a proprietary enzymatic assay in combination with a most probable number (MPN) tray, which allows for analysis of up to 100 mL without the need for membrane filtration. Since Legiolert is specific to *L. pneumophila*, it theoretically does not require a confirmation step, but previous studies have shown that it is also susceptible to false positives due to non-target bacterial growth (24), particularly in groundwater (25). Confirmation can be achieved using a variety of methods such as demonstrating lack of growth in the absence of L-cysteine (as recommended by ISO Method 11731:2017 [26]), latex agglutination, or with more advanced techniques such as matrix-assisted laser desorption/ionization mass spectrometry (MALDI-MS).

As an alternative to culture methods, relevant DNA targets can be detected and quantified via quantitative polymerase chain reaction (qPCR), droplet digital PCR (ddPCR), or digital PCR (dPCR). These molecular assays can potentially be completed within hours and can be tailored to *Legionella* spp. via the *ssRA* gene (27), *L. pneumophila* via the *mip* gene (28), and even to *L. pneumophila* serogroup (SG) 1 via the *wzm* or *srkA* gene (29, 30). SG1-specific assays are of particular value in a public health context since SG1 has been implicated in many documented outbreaks (8, 31). While molecular methods can potentially provide highly specific counts, encompassing both culturable and VBNC organisms, they can also capture non-viable organisms that are unlikely to pose a public health concern, potentially confounding any subsequent utility, regulatory, or public health action. Molecular techniques also require specialized equipment, skilled technicians, and potentially costly consumables relative to culture methods.

In support of the recently developed NDEP guidance, the objective of this study was to further substantiate *L. pneumophila* occurrence in Southern Nevada groundwater by evaluating several confirmation methodologies following initial detection and quantification by Legiolert, as it was expected that Legiolert may suffer from a high false positive rate when used for groundwater (25). Using liquid media harvested from Legiolert-positive trays, confirmation methods included latex agglutination, MALDI-MS, “rapid” qPCR (*mip* and *srkA* genes), and traditional plating on BCYE agar (±L-cysteine), with the first three methods able to achieve confirmation in <24 h. The results from this study improve our understanding of *L. pneumophila* occurrence in groundwater, support future operational decision-making efforts by SNWA and other drinking water systems, and provide pertinent information related to false positives, including identification of interfering organisms.

## MATERIALS AND METHODS

### Sample collection and initial analysis by Legiolert

Samples were collected from groundwater wells that were either discharging to the distribution system (direct-to-distribution system wells, DDSW) or discharging to chlorinated reservoirs (reservoir wells, RW) across the Las Vegas Valley (Nevada, USA). Samples were collected during a 2-month period encompassing well start-up and groundwater delivery to consumers (late April through early July 2025). During start-up, all wells were flushed until turbidity levels were <3 NTU. During routine operation, groundwater was dosed with sodium hypochlorite, either at the wellhead (DDSWs) or at the reservoir (RWs), to achieve a free chlorine residual of 1 mg/L; turbidities <1 NTU and free chlorine residuals >0.5 mg/L were targeted within the distribution system. The samples included in this confirmation study were collected from DDSWs and RWs at various stages (i.e., during start-up/clean-up and post-delivery) and with varying levels of turbidity and free chlorine (Table S1 in the Supplementary Information). Since this study includes only Legiolert-positive samples, a majority of the RW data represents samples collected at the wellhead prior to chlorination; water from reservoirs was entirely non-detect for *L. pneumophila* and, thus, omitted from this study.

Groundwater samples were collected directly into 150-mL sample bottles containing sodium thiosulfate (IDEXX, Westbrook, ME), transported on ice back to the laboratory where they were stored at 4°C and analyzed within 24 h using the Legiolert method, per manufacturer’s instructions (IDEXX). Briefly, hardness levels were checked using hardness test strips provided with the IDEXX Legiolert Supplement Kit (Aquadur, Macherey-Nagel, Germany), and appropriate levels of Legiolert supplement (IDEXX) were added prior to the addition of Legiolert substrate (IDEXX). Select early season groundwater samples with higher turbidities, diluted using autoclaved milliQ water, per manufacturer’s instructions, to analyze 10 mL and/or 1 mL sample volumes. Prepared samples were poured into MPN well trays (IDEXX) and incubated at 39°C for 7 days. For any positive samples, ~3 mL of Legiolert media was extracted with a 25G needle from a single large positive well and immediately confirmed for *L. pneumophila*. If a large well was unavailable, ~0.3 mL was collected from a small positive well and immediately confirmed for *L. pneumophila*; however, this limited the ability to perform all of the confirmation techniques, specifically MALDI-MS.

### Confirmation methods

**Fig 1:**
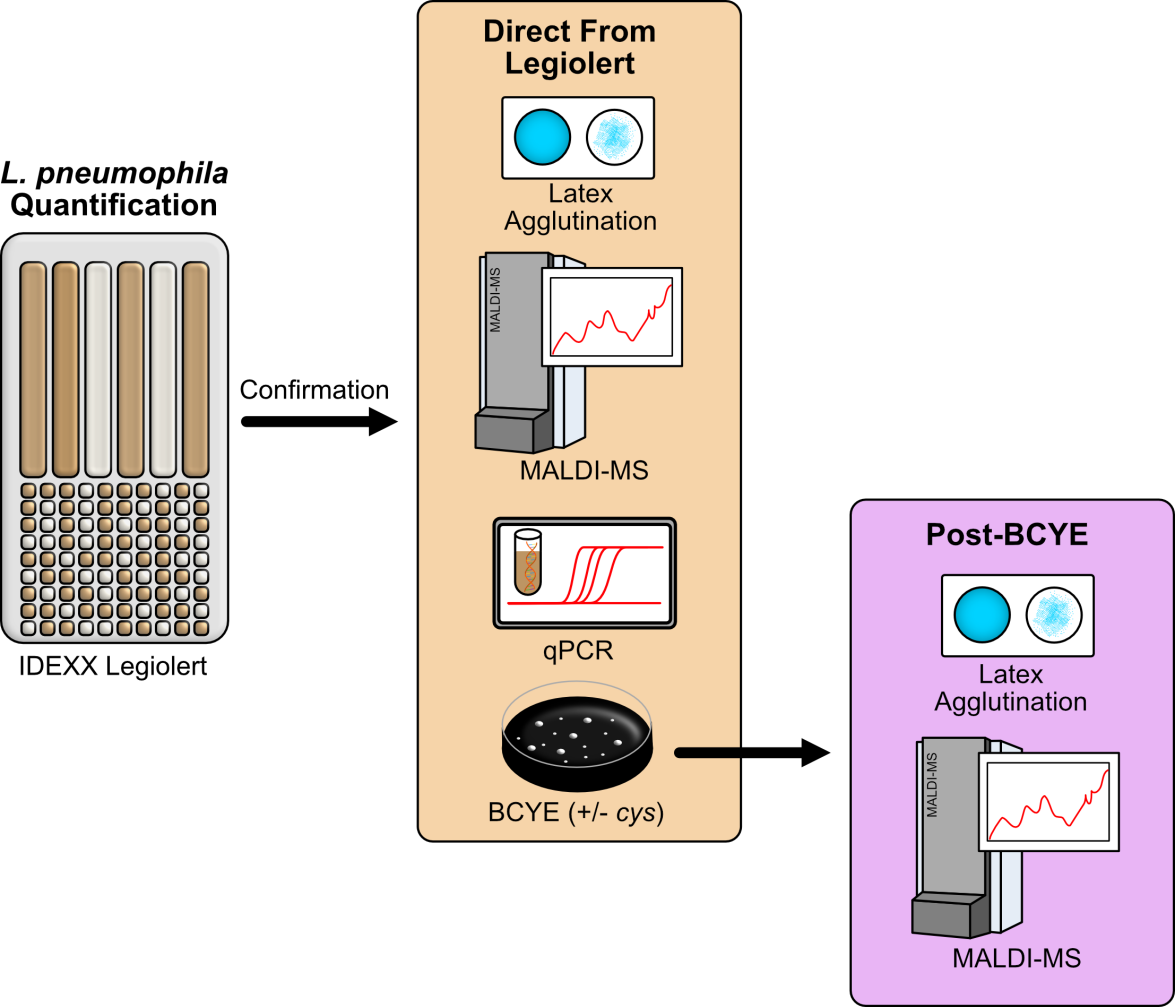
Overview of *L. pneumophila* confirmation comparison workflow.

As described in the following sections, confirmation of positive Legiolert wells was performed via latex agglutination, MALDI-MS, rapid qPCR, and plating on BCYE agar (±L-cysteine). To assess potential incompatibility with Legiolert media, confirmation via latex agglutination and MALDI-MS was performed directly from Legiolert-positive media and indirectly with a single colony grown from Legiolert-positive media on BCYE agar (+L-cysteine). An overview of the confirmation method workflow is provided in Fig. 1.

#### Latex agglutination

For confirmation and serogroup determination, latex agglutination was performed using the Oxoid Legionella Latex Test kit (Oxoid Ltd., United Kingdom). For direct analysis from Legiolert-positive trays, Legiolert media was collected from a positive well with a 25G needle, and one or two drops were separately mixed with SG1 latex beads, SG2-14 latex beads, and non-pneumophila Legionella latex beads. For analysis from BCYE plates, a colony that was morphologically consistent with *L. pneumophila* (when present) was collected with a loop and mixed with a drop of Oxoid Legionella Suspension Buffer. Then, the suspended colony was subsequently mixed with SG1, SG2-14, and non-pneumophila Legionella beads. After mixing with the respective latex beads, samples were rocked for 2 min, and then agglutination results were recorded. If no agglutination occurred, the sample was designated as “No Reaction.” Controls were included following manufacturer’s instructions.

#### MALDI-MS

MALDI-MS confirmation was performed using a MALDI Biotyper sirius (Bruker Scientific, Germany). When confirming directly from Legiolert, 1 mL of liquid media from a positive well was transferred to a microcentrifuge tube, spun down at 13,000 × *g* for 2 min, and resuspended in 1 mL of LC/MS grade water; the centrifugation/resuspension step was repeated one additional time with a final resuspension volume of 300 µL. Then, the tubes were amended with 900 μL of 100% EtOH, mixed via aspiration, and centrifuged at 13,000 × *g* for 2 min. The supernatant was removed by pipette, the sample was re-centrifuged at 13,000 × *g* for 2 min, and any remaining supernatant was once again removed by pipette, followed by air drying for at least 5 min. The pellet was then resuspended in 25 μL of 70% formic acid via aspiration, amended with 25 μL of 100% acetonitrile followed by mixing via aspiration, and then centrifuged one final time at 13,000 × *g* for 2 min. One microliter of the supernatant was spotted onto the MALDI target plate (in duplicate) and allowed to dry, and then 1 μL of 10 mg/mL 2-Cyano-3-(4-hydroxyphenyl)acrylic acid (US IVD HCCA Matrix Solution, Bruker) was applied to each sample spot. For colonies isolated from BCYE agar (+L-cysteine), a toothpick was used to transfer a small amount of a single colony onto the target plate (in duplicate). One microliter of 70% formic acid was applied to the sample spot, followed by 1 μL US IVD HCCA Matrix Solution, allowing each to dry. The plates were then analyzed using the MBT Compass GT software (Bruker Scientific, Germany). *L. pneumophila* identification was considered confirmed when at least one spot per duplicate returned a high confidence score (log score >2.00). For quality control, Bruker US IVD Bacterial Test Standard, prepared per manufacturer’s instruction, was analyzed with each run.

#### Rapid qPCR

Confirmation by “rapid” qPCR was performed only on liquid media harvested directly from Legiolert-positive wells. One milliliter of media was transferred to a microcentrifuge tube, centrifuged at 13,000 × *g* for 2 min, and resuspended in 200 μL of molecular grade water. To reduce workload and obtain more rapid results, DNA extraction was performed using a heat treatment method (32–34) instead of a kit-based extraction approach. Briefly, the tubes were heated at 95°C for 10 min followed by a quick spin down and mixing via aspiration. The heat-treated samples were analyzed by qPCR using a CFX384 Touch Real-Time PCR Detection System (Bio-Rad, Hercules, CA) for the following two gene targets: *mip* (identification of *L. pneumophila*) and *srkA* (identification of *L. pneumophila* SG1) (30, 35) (Table S2). Ten microliter reactions were prepared with 1 µL of nucleic acid template (i.e., heat-treated sample), 5 μL of either Bio-Rad SsoAdvanced Universal Probes Supermix (*mip*) or Bio-Rad iTaq Universal SYBR Green Supermix (*srkA*), 0.4 μL each of the forward and reverse primers (final concentrations of 20 µM), 0.25 µL of the 20 µM probe (*mip* only), and approximately 3 µL of molecular grade water. Cycling conditions for each assay are provided in Table S2. The presence of the *mip* gene via qPCR was assumed to be indicative of enriched *L. pneumophila* within a positive Legiolert tray; this was used as a baseline confirmation determination (i.e., true positive) for comparison with the other confirmation techniques.

#### Buffered charcoal yeast extract (±L-cysteine)

Liquid media harvested from Legiolert-positive wells was carefully streaked onto BCYE agar with and without L-cysteine, a necessary amino acid for the growth of *L. pneumophila*. The growth on BCYE (+L-cysteine) in combination with the inability to grow on BCYE (−L-cysteine) indicates the likely presence of *L. pneumophila* in the original sample (ISO).

### Statistical analysis

All statistical analyses were performed with R (v 4.5.0). One-way analysis of variance (ANOVA) was performed to test for significant differences (*α* = 0.05) across measured water quality parameters (*L. pneumophila* concentration, turbidity, and free chlorine concentration). If significant differences were determined, Tukey’s Range Test was performed to determine which conditions were influenced by the specified water quality parameter. Water quality data that were below the limit of detection (LOD) were substituted with LOD/sqrt (2).

## RESULTS

### Overview of samples

During the 2025 well season, >600 groundwater samples were collected during initial cleanup/flushing, post-cleanup, and post-chlorination (including during production). One hundred five unique samples, determined to be presumptive positive for *L. pneumophila* via Legiolert, were randomly selected for confirmation testing, resulting in 108 positive Legiolert assays (2 samples were assayed using multiple dilutions) (Table S1). Of the 105 unique samples, 40% were associated with DDSW samples and the remaining 60% with RW samples, although a majority of the RW samples were collected prior to chlorination. Sample turbidities ranged from <0.3 to 35.6 NTU for DDSW samples and from <0.3 to 2.95 NTU for RW samples, and free chlorine residuals ranged from <0.04 to 2.40 mg/L for DDSW samples and from <0.04 to 6.20 mg/L for RW samples. The highest free chlorine residuals were associated with one particular groundwater well with frequent *L. pneumophila* detections, thus prompting these augmented disinfection measures. *L. pneumophila* concentrations ranged from 1 to 689 MPN per 100 mL (Fig. S1), with a median of 4.0 MPN/100 mL. Without additional treatment (i.e., flushing, chlorination, and/or UV disinfection), 43 samples would have exceeded NDEP’s treatment-based goal of 5 MPN/100 mL, and 12 samples would have exceeded the NDEP and CDC action level of 100 MPN/100 mL. The 43 samples exceeding 5 MPN/100 mL were collected across 19 different wells prior to the groundwater entering the distribution system or a reservoir. Ten of these wells fed reservoirs (i.e., RWs), so the groundwater was ultimately exposed to extended chlorine Cts prior to distribution. The remaining nine were DDSWs and, thus, prompted a variety of operational responses in accordance with the NDEP guidelines, including sample tap or well disinfection followed by repeat sampling (six of nine DDSWs) or even well shutdown (four of nine DDSWs). Notably, one post-flushing sample from a particular well returned a *L. pneumophila* concentration of 106 MPN/100 mL. Despite a repeat sample and corresponding distribution system sample returning concentrations of <1 MPN/100 mL, this well was proactively shutdown for the season. The initial result was ultimately characterized as a false positive, as described later.

### Confirmation results

**Fig 2:**
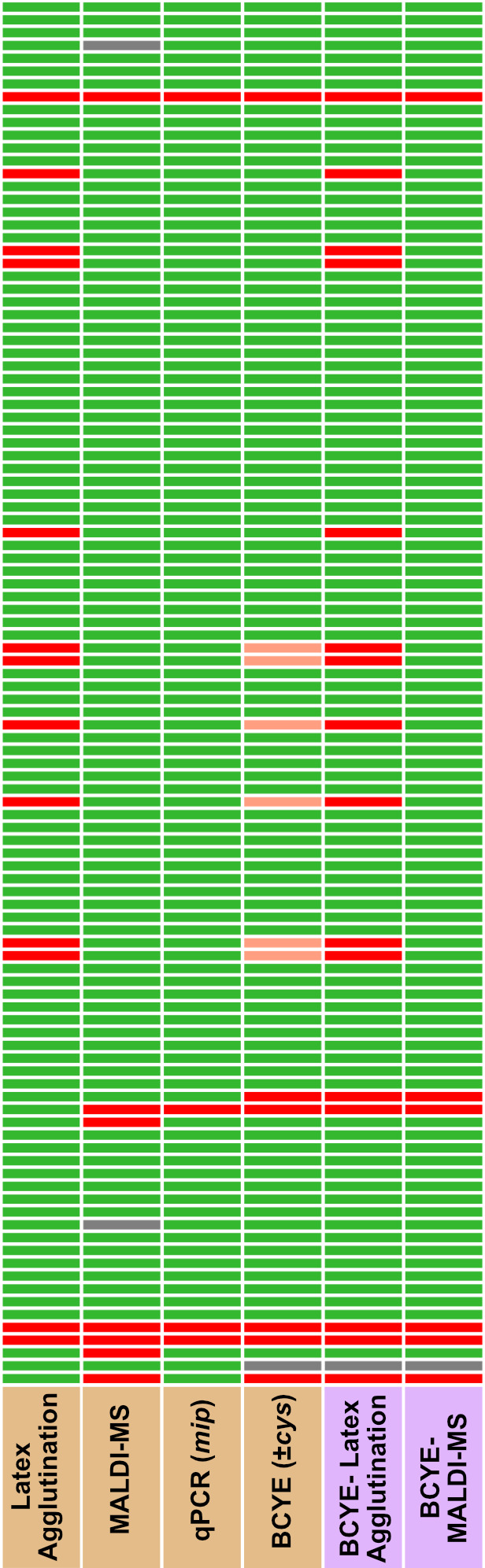
Heat map of *L. pneumophila* confirmation results; each row represents 1 of 108 samples listed in chronological order of collection (bottom to top). Green = confirmed as *L. pneumophila*; red = not confirmed as *L. pneumophila*; light red (BCYE column) = confirmed as *L. pneumophila* by qPCR/MALDI-MS with growth observed on BCYE -L-cysteine; gray (MALDI-MS column) = insufficient vol for test; gray (BCYE columns) = fungal overgrowth. See Table S1 for detailed sample descriptions. MALDI-MS: Matrix-assisted laser desorption/ionization mass spectrometry. qPCR (mip): quantitative polymerase chain reaction of the macrophage infection potentiator gene. BCYE (+/−cys): Buffered-charcoal yeast extract with and without L-cysteine.

Direct confirmation results are summarized in Table 1, and a more detailed sample characterization is provided in Table S1. A heat map overview of each sample is also provided in Fig. 2. The following sections are divided by method, with further distinction for confirmation methods that have the potential to provide results in minutes to hours following Legiolert enrichment (<24 h; latex agglutination, MALDI-MS, qPCR) vs the methods requiring ≥48 h to confirmation (BCYE, BCYE + latex agglutination, BCYE + MALDI-MS). Confirmation results were considered accurate when there was agreement with qPCR results, with the exception of the two samples analyzed with dilutions (Table S1).

**TABLE 1:** Direct *L. pneumophila* (Lp) confirmation results from Legiolert-positive trays.^*a*^

*n*	Method	Lp positive (%)	Lp negative or ND (%)	Serogroup (SG)		
				SG1	SG2-14	ND
108	Latex Agglutination	95 (88%)	13 (12%)	43	52	13
	MALDI-MS	99 (92%)	9 (8%)	NA	NA	NA
	qPCR	104 (96%)	4 (4%)	40	64	4
	BCYE (+/*−cys*)	95 (88%)	13 (12%)	NA	NA	NA

^*a*^ NA = not applicable; ND = non-detect or not determined.

#### Confirmation by direct latex agglutination (<24 h)

Latex agglutination was able to confirm *L. pneumophila* and provide a serogroup determination in 95 of 108 (88%) Legiolert-positive trays, with 43 of the 95 (45%) confirmations reacting with SG1 beads (Table 1). For one of the two groundwater samples analyzed using multiple volumes (i.e., 1, 10, and 100-mL of groundwater), latex agglutination confirmed *L. pneumophila* SG1 when 1 mL of sample volume was analyzed by Legiolert, but no reaction was observed for the 10-mL and 100-mL volumes (Table S1). The 10-mL and 100-mL sample volumes were similarly inconclusive with MALDI-MS, qPCR, direct BCYE (±L-cysteine), and the indirect BCYE methods, suggesting a potential interference in the larger sample volumes. On the other hand, all direct and indirect approaches (with the exception of direct MALDI-MS due to volume limitations) confirmed the *L. pneumophila* SG1 determination for the 1-mL sample volume. Therefore, confirmation with only the larger sample volumes would have resulted in an erroneous false positive determination since the 1-mL volume indicated the presence of *L. pneumophila* SG1 across all methods except MALDI-MS (no identification).

No reaction was observed with any of the latex agglutination beads in 11 additional samples. Of the 13 total “No RXN” results, 12 were ultimately determined to be false negatives, and an additional three SG1 determinations were characterized as erroneous when compared against corresponding results from the two qPCR assays. Thus, latex agglutination accurately confirmed *L. pneumophila* and its serogroup in 93 of 108 (86%) Legiolert-positive trays.

#### Confirmation by direct MALDI-MS (<24 h)

MALDI-MS was able to confirm *L. pneumophila* in 99/108 (92%) Legiolert-positive trays, although no serogroup determination was possible (Table 1; Table S1). Two were unable to be analyzed by MALDI-MS due to insufficient sample volumes from the Legiolert tray. Direct MALDI-MS was also unable to confirm or characterize any of the volumes analyzed for the three-dilution sample. *Indirect* MALDI-MS confirmation following isolation on BCYE (+L-cysteine) was successful for the 1-mL volume, but the 10-mL volume yielded a “No ID” result. Despite not being able to confirm *L. pneumophila* in the 100-mL volume, indirect MALDI-MS resulted in the detection of *Nocardia cyriacigeorgica* as a potentially interfering organism. While *Nocardia* spp. are commonly known for being filamentous, foam-causing, nuisance organisms in wastewater treatment applications, *N. cyriacigeorgica* is an emerging opportunistic bacterial pathogen associated with nosocomial infections of multiple organ systems, particularly the lungs (36). For the other sample analyzed in two volume aliquots, MALDI-MS was able to identify *L. pneumophila* in the 100-mL volume but was unable to confirm the 10-mL volume with direct or indirect MALDI-MS (despite confirmation via latex agglutination and qPCR).

There were three additional samples unable to be confirmed by MALDI-MS despite initial Legiolert positivity. Two of those samples resulted in “No ID” by MALDI-MS, with one being identified as *L. pneumophila* SG2-14 by all other confirmation methods and the other ultimately characterized as a Legiolert false positive based on latex agglutination and qPCR. For the latter, we still characterized the MALDI-MS result as inconclusive since there was no specific organism identified. For the third non-confirmed sample, MALDI-MS identified *Stenotrophomonas maltophilia*, which is another emerging opportunistic bacterial pathogen associated with nosocomial and community-acquired infections (37). As mentioned earlier, Legiolert initially indicated that this particular sample contained *L. pneumophila* at a concentration of 106 MPN/100 mL, prompting the season-long shutdown of the corresponding groundwater well before it was put into production mode.

#### Confirmation by rapid qPCR (<24 h)

qPCR of the *mip* gene was able to confirm *L. pneumophila* in 104/108 (96%) Legiolert-positive trays (Table 1), although two of the non-confirmed trays were ultimately characterized as Legiolert false positives. The two “false negatives” via qPCR of the *mip* gene were again related to the sample that was initially analyzed with three different volumes, with the 1-mL volume positive for the *mip* gene and the 10-mL and 100-mL volumes negative for the *mip* gene, and this was potentially due to competition from interfering organisms, though further investigation would be necessary to definitively conclude this outcome.

The additional qPCR assay for the *srkA* gene was able to determine that 40/108 (37%) Legiolert-positive trays contained *L. pneumophila* SG1. As mentioned earlier, the latex agglutination SG1 beads reacted for 43/108 (40%) trays. The latex agglutination SG1 total included one sample that failed to confirm as *L. pneumophila* by both MALDI-MS and qPCR, thus suggesting a false positive result by Legiolert and latex agglutination. An additional sample confirmed as SG1 via latex agglutination, but the *srkA* qPCR assay failed to amplify, suggesting a latex agglutination SG1 false positive. This was subsequently confirmed via indirect latex agglutination, which instead identified *L. pneumophila* SG2-14. The third discrepancy was a sample that confirmed positive for SG1 via direct and indirect latex agglutination, but the *srkA* qPCR assay failed to amplify, suggesting either a SG1 false negative via qPCR or potentially cross-reactivity with the SG1 latex beads. There has been demonstrated cross-reactivity with the SG1 beads by *L. pneumophila* SG9, per information provided in the Oxoid Legionella Latex Test instructions.

#### Confirmation by direct BCYE (±L-cysteine) and indirect latex agglutination or MALDI-MS (≥48 h)

BCYE agar (±L-cysteine) was able to confirm 95/108 (88%) Legiolert-positive trays (Table 1). Specifically, these 95 trays resulted in growth consistent with *L. pneumophila* morphology on the +L-cysteine plates coupled with no growth on the corresponding −L-cysteine plates. Of the 13 remaining samples, 12 yielded growth on the −L-cysteine plates, thus indicating false positives. Of these 12, 8 were confirmed as *L. pneumophila* via qPCR of the *mip* gene, and 7 were confirmed by MALDI-MS (Fig. 2; Table S1). One of the 13 samples was unable to be confirmed by BCYE due to fungal overgrowth. Thus, BCYE confirmation was determined to have accurately identified *L. pneumophila* in only 90% of the Legiolert-positive trays, which exceeded only latex agglutination (86% accurate). Interestingly, 6 of the 7 samples that grew on BCYE (−L-cysteine) but were confirmed by qPCR and MALDI-MS had growth consistent with *L. pneumophila* morphology but grew at a much slower rate and lower density. Colonies from these plates were regrown on fresh BCYE (−L-cysteine) to ensure that this was not an artifact of L-cysteine carryover from the Legiolert tray; growth was consistent even on the new plates. All six of these samples failed to react with latex agglutination but were determined to be SG2-14 based on qPCR (i.e., positive for *mip* and negative for *srkA*).

Since latex agglutination and MALDI-MS are not necessarily meant to be used in conjunction with Legiolert media, it was hypothesized that indirect confirmation (i.e., coupling BCYE growth with subsequent latex agglutination or MALDI-MS) might yield better results. Following isolation on BCYE (+L-cysteine), latex agglutination was able to confirm 91/108 (84%) samples, while MALDI-MS evaluation of the same colonies was able to confirm 101/108 (94%) samples (Table 2). This represents a slightly lower confirmation rate for indirect vs direct latex agglutination and a slightly higher confirmation rate for indirect vs direct MALDI-MS. Also, indirect latex agglutination determined that 38 of 91 (42%) confirmed samples belonged to SG1, which was also slightly lower than the 43 of 95 (45%) samples confirmed by direct latex agglutination as SG1. Although indirect MALDI-MS led to the identification of a co-occurring organism (*N. cyriacigeorgica*) in one additional sample, there were no other major benefits of BCYE isolation prior to latex agglutination or MALDI-MS. The aforementioned identification of *Stenotrophomonas maltophilia* was achieved by both direct and indirect MALDI-MS.

**TABLE 2:** Indirect *L. pneumophila* (Lp) confirmation results from Legiolert-postive trays (i.e., after isolation on BCYE [+*cys*]). ND = non-detect or not determined, NA = not applicable

*n*	Method	Lp positive (%)	Lp negative or ND (%)	Serogroup (SG)		
				SG1	SG2-14	ND
108	Latex Agglutination	91 (84%)	17	38	53	17
	MALDI-MS	101 (94%)	7	NA	NA	NA

### Influence of groundwater quality

**Fig 3:**
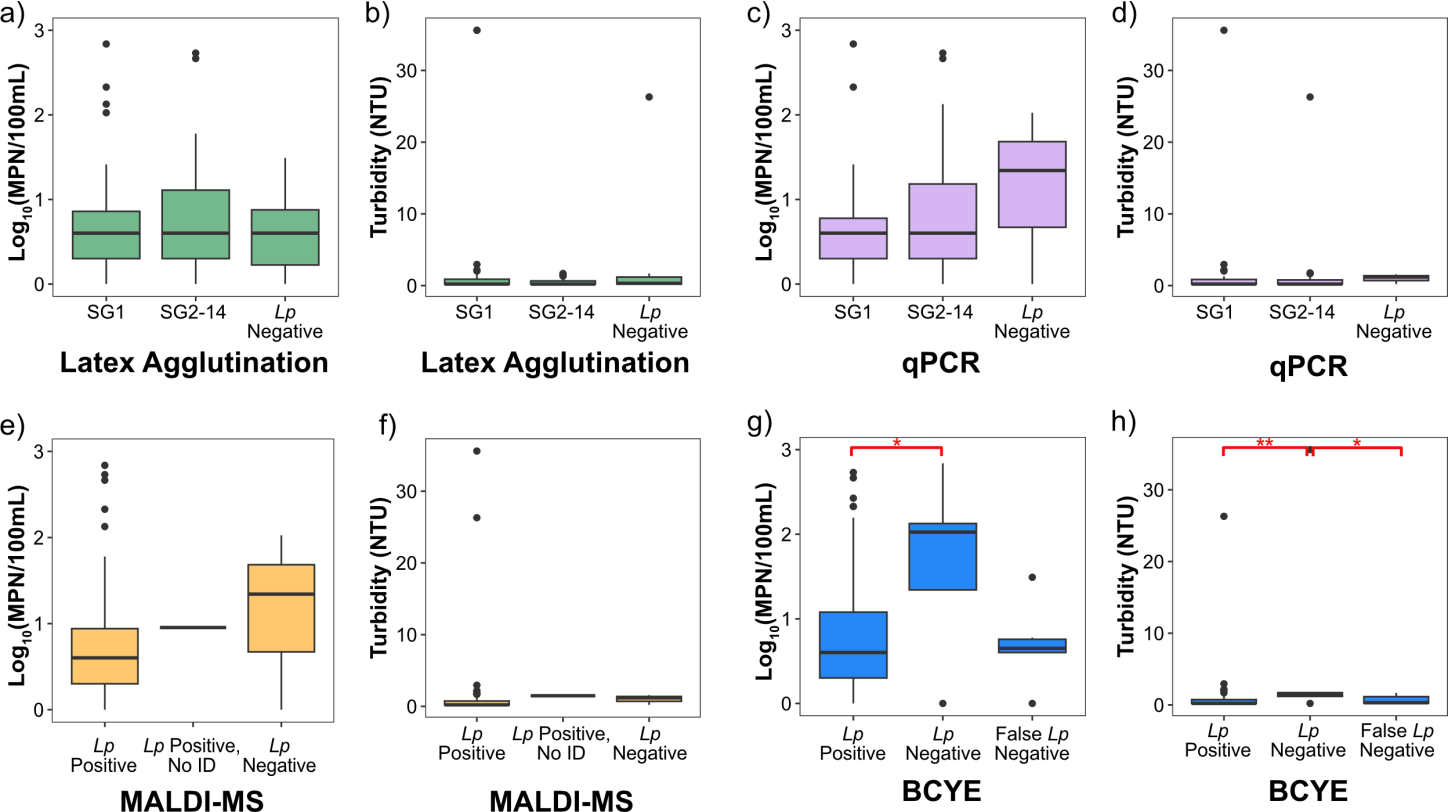
Influence of *L. pneumophila* concentration and turbidity in original samples on confirmation by (**a, b**) latex agglutination, (**c, d**) qPCR, (**e, f**) direct MALDI-MS, and (**g, h**) BCYE. For MALDI-MS, “*Lp* Positive, No ID” refers to samples that were unable to be confirmed by MALDI-MS but were positive for the *mip* gene by qPCR. For BCYE, “False *Lp* Negative” refers to samples that supported growth on −L-cysteine plates (i.e., negative) but were otherwise confirmed positive for *L. pneumophila*. *P < 0.05, **P < 0.005.

Groundwater samples were collected at multiple locations and across the duration of the well start-up period, capturing a range of free chlorine, turbidity, and *L. pneumophila* levels. Mean free chlorine concentrations at the time of sampling were not significantly different across the groupings for each confirmation method (ANOVA *P* > 0.05) (data not shown; groupings shown in Fig. 3). Similarly, mean turbidity and *L. pneumophila* concentration were not significantly different across the SG1, SG2-14, and non-confirmed groupings for direct latex agglutination and qPCR, nor across samples that were or were not able to be identified as *L. pneumophila* via the Bruker BDAL Library during MALDI-MS (ANOVA *P* >0.05) (Fig. 3a through e).

Confirmation via plating on BCYE (±L-cysteine) exhibited significant differences with respect to *L. pneumophila* concentrations and turbidity (Fig. 3g and h). Samples that failed to confirm by BCYE had significantly higher turbidities (*P* <0.05) than samples that confirmed positive or were deemed “false negative,” which refers to samples that supported growth on BCYE (−L-cysteine) but were otherwise confirmed positive for *L. pneumophila*. Similarly, samples that failed to confirm by BCYE appeared to have significantly higher concentrations of *L. pneumophila* (*P* <0.05). Nevertheless, a greater number of samples covering a range of water quality parameters is needed to confidently characterize water quality impacts on various confirmation approaches. However, this analysis still demonstrates that there are differences between approaches that may be exacerbated by water quality, potentially justifying a multi-pronged confirmation approach for high-consequence monitoring applications.

## DISCUSSION

Despite the Legiolert method being selective for *L. pneumophila*, there is still a need for confirmation, as demonstrated here and elsewhere (24, 25). Of the 108 Legiolert-positive trays in this study, three were unable to be confirmed as *L. pneumophila* by any confirmation method, with two of these coming from different dilutions of the same initial sample. Additionally, there was a sample that was confirmed as *L. pneumophila* SG1 by latex agglutination but failed to confirm by the remainder of the methods. This was the only instance in which qPCR and MALDI-MS were unable to confirm a sample that appeared to react with the latex agglutination SG1 beads. However, this may be due to the subjective nature of the latex agglutination test, as the results rely on the analyst’s interpretation of agglutination. There is also potential that the sample matrix was able to cross-react with the latex agglutination SG1 beads. Once the same sample was re-analyzed after isolation on BCYE, there was no longer any reactivity with the latex agglutination beads. MALDI-MS confirmed the presence of *S. maltophilia* in both the liquid taken from the Legiolert tray (direct MALDI-MS) and the BCYE isolate (indirect MALDI-MS). *Stenotrophomonas* spp. has been previously associated with cross-reactivity with Legiolert (25). This sample, which was ultimately classified as a false positive, initially resulted in a Legiolert concentration of 106 MPN/100 mL, exceeding the 100 MPN/100 mL action level identified by NDEP and the CDC. As noted earlier, this led to the proactive shutdown of that particular well despite follow-up sampling associated with that location returning non-detects by Legiolert.

The method with the highest rate of confirmed *L. pneumophila* was direct qPCR of the *mip* gene (96%), followed by MALDI-MS (92%), latex agglutination (88%), and BCYE (±L-Cysteine) (88%). Each of these potential confirmation methods utilizes different bacterial characteristics to confirm the presence of *L. pneumophila*, which might contribute to differing efficacy. BCYE focuses on the metabolic requirements of *L. pneumophila,* specifically its inability to grow on growth medium without L-cysteine supplementation. However, in this study, we were able to isolate six samples in which *L. pneumophila*, as confirmed by MALDI-MS and qPCR, was able to grow on BCYE (−L-cysteine), albeit at lighter growth density than typical, highlighting a weakness of this method. Weak growth on BCYE (−L-cysteine) has been demonstrated for *Legionella oakridgensis* and *Legionella spiritensis* (26), but there appear to be no described instances of *L. pneumophila* growing on BCYE (−L-cysteine) in the literature. As previously mentioned, the samples that produced weak growth on BCYE (−L-cysteine) were also unable to react with either set of latex agglutination beads (SG1 or SG2-14) but were determined to be SG2-14 based on qPCR (+*mip* and *−srkA*). These isolates may represent a strain of *L. pneumophila* that has yet to be described, warranting further investigation. Because of this shortcoming, this method should not be implemented for confirmation within the study context. An additional major drawback for the use of BCYE (±L-cysteine) is the associated incubation time, which can range from 2 to 10 days. When used for confirmation (i.e., Legiolert followed by BCYE), the combined incubation time could require more than 2 weeks. Despite these analytical shortcomings, BCYE (±L-cysteine) is the least analyst-intensive and least expensive method for *L. pneumophila* confirmation and the only confirmation method in this study that directly infers viability (though Legiolert is also a viability-based quantification technique) (Table 3).

**TABLE 3:** Overview of *L. pneumophila* confirmation methods

Method	Equipment needed	Cost	Time to result^*a*^	Serogroup determination
Latex Agglutination	Beads/Kit	$	<10 min	Yes (SG1 vs SG2-14)
MALDI-MS	Specialized Instrument	$$$	≤1 h	Not currently, though there is potential with custom database (38)
qPCR (*mip*)	Specialized Instrument	$$	2–3 h	No, but alternative assay (*srkA* gene) can distinguish SG1
BCYE (±L-cysteine)	Incubator	$	2–7 days	No, but can be coupled with indirect latex agglutination (additional ~10 min)

^*a*^ Time to result is post-Legiolert (i.e., 7 days of incubation prior to confirmation).

Latex agglutination relies on reactivity of the O-antigen group of the lipopolysaccharide region of the *L. pneumophila* cell membrane. These kits contain agglutination beads with antibodies specific to either SG1 isolates, SG2-14 isolates, and select non-pneumophila *Legionella* species. The results from this study indicate that there exist non-SG1 *L. pneumophila* isolates that are unable to react with the SG2-14 antibodies utilized in the Oxoid Latex Agglutination Test kit. This was the case for 10 of 13 positive samples (i.e., able to be confirmed by MALDI-MS and/or qPCR) that were unable to react with the latex agglutination beads. In addition to its higher inaccuracy rate, this method also relies heavily on the subjectivity of the analyst. Despite these drawbacks, it is a relatively inexpensive and rapid test that requires very little sample volume (Table 3).

MALDI-MS had the second highest *L. pneumophila* confirmation rate. MALDI-MS relies on the protein “fingerprint” of a bacterial cell, which generally has sufficient resolution to differentiate at the species level. However, identification relies on the availability of a mass spectra database for cross-referencing generated spectra. In this case, the database was supplied and regularly updated by the manufacturer (Bruker Scientific, Germany) within the instrument software, although it is also possible for users to create their own database. In this study, three samples were confirmed as *L. pneumophila* via the presence of the *mip* gene but were unable to be confirmed with the existing Bruker database. It is unclear whether this was driven by water quality impacts or whether this could potentially be addressed with updates to the database. Nevertheless, MALDI-MS is a promising tool that can not only identify *L. pneumophila* for confirmation purposes but can also rapidly identify interfering/cross-reacting organisms, some of which may pose similar public health risks. MALDI-MS does require expensive equipment and consumables (Table 3), has not yet been widely implemented by water utilities, and is generally limited to species-level identification, although there is some evidence of serogroup identification capabilities (38).

The method with the highest rate of *L. pneumophila* confirmation was qPCR of the *mip* gene. This confirmation method is based on the presence of specific genetic material contained within the cell, which in this case is highly conserved among *L. pneumophila* strains. qPCR also allows for additional assays to be administered on the same sample, such as for the *srkA* gene to confirm SG1, or perhaps for other opportunistic pathogens relevant to a given application (e.g., groundwater monitoring). PCR-based methods provide rapid results but do require specialized equipment, reagents, and trained analysts (Table 3). However, many water quality labs already employ some sort of PCR-based methodology, and the addition of published assays to current workflows is straightforward. Although not addressed in the current study, the rapid qPCR approach could be further expanded and/or streamlined by implementing multiplex assays (e.g., simultaneous analysis of the *ssRA*, *mip*, and *srkA/wzm* genes).

### Study limitations

There are several limitations to the current study. For all samples, only a single Legiolert well was analyzed for *L. pneumophila* confirmation. Previous studies have indicated that individual wells may harbor different *L. pneumophila* strains (39). Therefore, there is potential for different wells to harbor cross-reacting bacterial species while still containing *L. pneumophila* in other wells. To test this potential, every large positive well and ~50% of the small positive wells from a single Legiolert tray were tested via qPCR (*mip* and *srkA*), and all resulted in positive detection of *L. pneumophila* SG1. Regardless, it is recommended that multiple wells be confirmed for each tray, or if a selected well fails to confirm, additional wells should be tested prior to making a “false positive” determination. How results should be reported in these instances is not described by IDEXX, but perhaps a solution akin to the ISO method, which involves a correction factor (e.g., $\frac{fraction\;positive\;confirmed}{fraction\;total\;confirmation\;tested}$), could be integrated prior to MPN calculation.

The goal of this study was to focus on confirmation of the Legiolert method for groundwater samples, as groundwater has been shown to have a higher false positive rate than surface water samples (25) and is a common drinking water source in many regions. Further work evaluating confirmation methods for Legiolert across different water matrices is warranted, particularly for non-potable environmental matrices that are also known to harbor *L. pneumophila* and could present a public health risk (e.g., cooling towers, recycled water, hot springs, etc.). Moreover, the current study focused exclusively on Legiolert-positive trays, so it does not provide any information on the potential for false negatives, and thus sensitivity and specificity, when using Legiolert for groundwater monitoring. Generally, the 100-mL Legiolert test has been previously shown to exhibit high (>90%) specificity (i.e., low false positive rate) (25, 40, 41) compared to direct plating for potable water samples from both surface and groundwater sources. However, the sensitivity of the 100-mL Legiolert test appears to range anywhere from 21% to 87%, with seemingly negative impacts due to water quality (25). Further evaluations of Legiolert performance for minimally treated groundwater and other non-potable waters need to be performed. It should be noted that Scaturro (41) also reported higher sensitivity for qPCR than for Legiolert (94%–98%), but they found that qPCR also suffers from variable and potentially low specificity (48%–98%), depending on extraction method utilized and specific gene target selection. Similarly, combining BCYE with MALDI-MS confirmation substantially reduced the false positive rate in Donohue et al. (25); however, 25% (42/167) of samples were determined to be false negatives, which can have a greater impact on public health. Each quantification technique has its drawbacks, and further studies on a wider array of water matrices would greatly benefit interpretation of results across a variety of water sources.

### Conclusion

Of all the confirmation methods evaluated in this study, qPCR of the *mip* gene was able to confirm *L. pneumophila* in positive Legiolert trays at the highest rate. Through the addition of the *srkA* assay, qPCR was also able to provide serogroup information (i.e., SG1 vs non-SG1). MALDI-MS had the second highest *L. pneumophila* confirmation rate, and MALDI-MS also demonstrated the added benefit of providing the identification of cross-reacting bacterial species, which could be further improved as databases are expanded and/or customized. Latex agglutination and BCYE (±L-cysteine) had the lowest *L. pneumophila* confirmation rate, and in one case in which the Legiolert concentration was >100 MPN/100 mL, latex agglutination erroneously confirmed *L. pneumophila* SG1, which prompted utility action. Indirect confirmation by isolating colonies on BCYE (+L-cysteine) prior to latex agglutination or MALDI-MS did not result in meaningful confirmation benefits. Overall, the Legiolert method appeared to have a low false positive rate (2%) for *L. pneumophila* in Southern Nevada groundwater samples. Although it is theoretically specific to *L. pneumophila*, which was also demonstrated in this study, confirmation still offers a number of benefits and should be encouraged, at least on samples where action levels have been exceeded and could inform subsequent actions.

## SUPPLEMENTAL MATERIAL

Supplemental tables and figure spectrum.01228-26-s0001.pdf Table S1: Full confirmation dataset. Table S2: qPCR assay details. Figure S1: Summary of Legiolert Concentrations.

ASM does not own the copyrights to Supplemental Material that may be linked to, or accessed through, an article. The authors have granted ASM a non-exclusive, world-wide license to publish the Supplemental Material files. Please contact the corresponding author directly for reuse.
