# Supplementary material for: Evaluation of Legionella pneumophila confirmation methods following IDEXX Legiolert analysis of groundwater
Source: Microbiol Spectr. 2026 Jun 30;14(8):e01228-26. doi: 10.1128/spectrum.01228-26 (PMC13436385; doi:10.1128/spectrum.01228-26)

Supplementary Information

Evaluation of *Legionella pneumophila* confirmation methods following IDEXX Legiolert analysis of groundwater

Christina M. Morrison[a]*, Phillip Wang[a], Gabriela Portillo-Chamul[a], John Monohan[a], Kai Chung[a], Daniel Gerrity[a,b]

[a] Southern Nevada Water Authority, P.O. Box 99954, Las Vegas, NV, 89193, USA

[b] University of Nevada, Las Vegas, Department of Civil & Environmental Engineering and Construction, Box 454015, 4505 S. Maryland Parkway, Las Vegas, NV 89154 USA

*Corresponding author. Email: christina.morrison@snwa.com

*Submitted to *ASM Microbiology Spectrum*

Table S1. Results for each of the 108 *Legionella pneumophila* (Lp)-positive analyses of groundwater samples collected from direct-to-distribution system wells (DDSW) or wells feeding reservoirs (RW); samples are listed in chronological order of collection (04/28/2025 – 07/01/2025). Initial water quality results included Lp concentration (from Legiolert), free chlorine residual at the time of sample collection, and turbidity. Confirmation was performed directly from Lp-positive Legiolert media using latex agglutination (LA), MALDI-MS, qPCR (*mip* and *srkA* genes), and BCYE plating [±L-cysteine (*cys*)]. Additional confirmation was performed by first replating Legiolert media on BCYE (+*cys*) and then confirming by LA and MALDI-MS. The far right column summarizes the sample determinations: true positive (true) with serogroup or false positive (false). As noted in the far left columns, two groundwater samples were originally analyzed by Legiolert using multiple volumes, yielding conflicting confirmation data. Red font indicates results assumed to be incorrect or inconclusive relative to the determinations in the far right column.

Well Type	Initial Water Quality Results			Confirmation Results								Legiolert Determination
				Direct from Legiolert						From BCYE (+*cys*)		
	Lp Conc. (MPN/100 mL)	Free Chlorine (mg/L Cl_2)	Turbidity (NTU)	LA	MALDI-MS	qPCR (*mip*)	qPCR (*srkA*)	BCYE (+*cys*)	BCYE (-*cys*)	LA	MALDI-MS	
DDSW	689	<0.04	35.6									
	Sample Volume in Legiolert Tray = 10 mL			SG1	No ID	+	+	Growth (atypical)	Growth	No RXN	No ID	True (SG1)[d]
	Sample Volume in Legiolert Tray = 100 mL			SG1	Lp	+	+	Overgrowth (fungi)	NA	NA	NA	
DDSW	22	<0.04	1.19									
	Sample Volume in Legiolert Tray = 1 mL			SG1	No ID	+	+	Growth	No Growth	SG1	Lp	True (SG1)[d]
	Sample Volume in Legiolert Tray = 10 mL			No RXN	No ID	–	–	Growth (atypical)	Growth	No RXN	No ID	
	Sample Volume in Legiolert Tray = 100 mL			No RXN	No ID	–	–	Growth (atypical)	Growth	No RXN	Not Lp[c]	
DDSW	4	<0.04	0.92	SG2-14	Lp	+	–	Growth	No Growth	SG2-14	Lp	True (SG2-14)
DDSW	26	<0.04	0.56	SG2-14	Lp	+	–	Growth	No Growth	SG2-14	Lp	True (SG2-14)
RW	8	<0.04	0.63	SG1	Lp	+	+	Growth	No Growth	SG1	Lp	True (SG1)
RW	53	<0.04	0.87	SG2-14	Lp	+	–	Growth	No Growth	SG2-14	Lp	True (SG2-14)
RW	4	<0.04	2.95	SG1	Lp	+	+	Growth	No Growth	SG1	Lp	True (SG1)
RW	1	<0.04	1.08	SG2-14	Lp	+	–	Growth	No Growth	SG2-14	Lp	True (SG2-14)
RW	1	<0.04	0.85	SG1	Lp	+	+	Growth	No Growth	SG1	Lp	True (SG1)
DDSW	1	1.78	0.89	SG1	NA[a]	+	+	Growth	No Growth	SG1	Lp	True (SG1)
RW	7	<0.04	0.86	SG1	Lp	+	+	Growth	No Growth	SG1	Lp	True (SG1)
RW	16	<0.04	1.69	SG2-14	Lp	+	–	Growth	No Growth	SG2-14	Lp	True (SG2-14)

Well Type	Initial Water Quality Results			Confirmation Results								Legiolert Determination
				Direct from Legiolert						From BCYE (+*cys*)		
	Lp Conc. (MPN/100 mL)	Free Chlorine (mg/L Cl_2)	Turbidity (NTU)	LA	MALDI-MS	qPCR (*mip*)	qPCR (*srkA*)	BCYE (+*cys*)	BCYE (-*cys*)	LA	MALDI-MS	
RW	19	<0.04	0.87	SG1	Lp	+	–	Growth	No Growth	SG2-14	Lp	True (SG2-14)
RW	4	<0.04	2.03	SG1	Lp	+	+	Growth	No Growth	SG1	Lp	True (SG1)
RW	28	<0.04	0.55	SG2-14	Lp	+	–	Growth	No Growth	SG2-14	Lp	True (SG2-14)
RW	7	<0.04	0.71	SG2-14	Lp	+	–	Growth	No Growth	SG2-14	Lp	True (SG2-14)
RW	42	<0.04	0.98	SG2-14	Lp	+	–	Growth	No Growth	SG2-14	Lp	True (SG2-14)
RW	9	<0.04	1.48	SG2-14	No ID	+	–	Growth	No Growth	SG2-14	Lp	True (SG2-14)
DDSW	106	1.81	1.57	SG1	Not Lp[b]	–	–	Growth (atypical)	Growth	No RXN	Not Lp[b]	False
DDSW	134	0.07	1.77	SG1	Lp	+	–	Growth (atypical)	Growth	No RXN	No ID	True (SG2-14)[d]
DDSW	538	1.13	0.92	SG2-14	Lp	+	–	Growth	No Growth	SG2-14	Lp	True (SG2-14)
RW	3	<0.04	<0.3	SG2-14	Lp	+	–	Growth	No Growth	SG2-14	Lp	True (SG2-14)
RW	4	<0.04	<0.3	SG2-14	Lp	+	–	Growth	No Growth	SG2-14	Lp	True (SG2-14)
RW	2	<0.04	<0.3	SG2-14	Lp	+	–	Growth	No Growth	SG2-14	Lp	True (SG2-14)
RW	2	<0.04	<0.3	SG1	Lp	+	+	Growth	No Growth	SG1	Lp	True (SG1)
RW	12	<0.04	<0.3	SG1	Lp	+	+	Growth	No Growth	SG1	Lp	True (SG1)
DDSW	17	0.05	1.18	SG1	Lp	+	+	Growth	No Growth	SG1	Lp	True (SG1)
DDSW	463	1.27	0.54	SG2-14	Lp	+	–	Growth	No Growth	SG2-14	Lp	True (SG2-14)
DDSW	2	1.70	1.32	SG2-14	Lp	+	–	Growth	No Growth	SG2-14	Lp	True (SG2-14)
DDSW	213	1.22	2.18	SG1	Lp	+	+	Growth	No Growth	SG1	Lp	True (SG1)
DDSW	4	1.17	1.44	No RXN	Lp	+	–	Growth	Light Growth	No RXN	Lp	True (SG2-14)[d]
DDSW	31	1.27	1.68	No RXN	Lp	+	–	Growth	Light Growth	No RXN	Lp	True (SG2-14)[d]
DDSW	5	1.94	0.60	SG2-14	Lp	+	–	Growth	No Growth	SG2-14	Lp	True (SG2-14)
DDSW	4	NA	NA	SG2-14	Lp	+	–	Growth	No Growth	SG2-14	Lp	True (SG2-14)
DDSW	213	NA	NA	SG2-14	Lp	+	–	Growth	No Growth	SG2-14	Lp	True (SG2-14)
RW	5	<0.04	<0.3	SG1	Lp	+	+	Growth	No Growth	SG1	Lp	True (SG1)
RW	2	<0.04	<0.3	SG2-14	Lp	+	–	Growth	No Growth	SG2-14	Lp	True (SG2-14)
RW	1	<0.04	<0.3	SG1	Lp	+	+	Growth	No Growth	SG1	Lp	True (SG1)

Well Type	Initial Water Quality Results			Confirmation Results								Legiolert Determination
				Direct from Legiolert						From BCYE (+*cys*)		
	Lp Conc. (MPN/100 mL)	Free Chlorine (mg/L Cl_2)	Turbidity (NTU)	LA	MALDI-MS	qPCR (*mip*)	qPCR (*srkA*)	BCYE (+*cys*)	BCYE (-*cys*)	LA	MALDI-MS	
DDSW	6	<0.04	NA	SG1	Lp	+	+	Growth	No Growth	SG1	Lp	True (SG1)
DDSW	26	2.40	NA	SG2-14	Lp	+	–	Growth	No Growth	SG2-14	Lp	True (SG2-14)
RW	1	<0.04	<0.3	SG2-14	Lp	+	–	Growth	No Growth	SG2-14	Lp	True (SG2-14)
RW	1	<0.04	<0.3	SG2-14	Lp	+	–	Growth	No Growth	SG2-14	Lp	True (SG2-14)
DDSW	1	0.35	<0.3	No RXN	Lp	+	–	Growth	Light Growth	No RXN	Lp	True (SG2-14)[d]
RW	2	<0.04	<0.3	SG2-14	Lp	+	–	Growth	No Growth	SG2-14	Lp	True (SG2-14)
RW	2	<0.04	<0.3	SG2-14	Lp	+	–	Growth	No Growth	SG2-14	Lp	True (SG2-14)
RW	1	<0.04	<0.3	SG1	Lp	+	+	Growth	No Growth	SG1	Lp	True (SG1)
RW	2	<0.04	<0.3	SG1	Lp	+	+	Growth	No Growth	SG1	Lp	True (SG1)
DDSW	5	0.05	<0.3	SG1	Lp	+	+	Growth	No Growth	SG1	Lp	True (SG1)
DDSW	4	1.39	0.32	No RXN	Lp	+	–	Growth	Light Growth	No RXN	Lp	True (SG2-14)[d]
RW	2	<0.04	<0.3	SG1	Lp	+	+	Growth	No Growth	SG1	Lp	True (SG1)
RW	2	<0.04	<0.3	SG2-14	Lp	+	–	Growth	No Growth	SG2-14	Lp	True (SG2-14)
RW	1	<0.04	1.15	SG2-14	Lp	+	–	Growth	No Growth	SG2-14	Lp	True (SG2-14)
RW	12	<0.04	<0.3	SG2-14	Lp	+	–	Growth	No Growth	SG2-14	Lp	True (SG2-14)
DDSW	5	1.07	0.30	No RXN	Lp	+	–	Growth	Light Growth	No RXN	Lp	True (SG2-14)[d]
DDSW	6	2.10	<0.3	No RXN	Lp	+	–	Growth	Light Growth	No RXN	Lp	True (SG2-14)[d]
RW	2	1.46	<0.3	SG2-14	Lp	+	–	Growth	No Growth	SG2-14	Lp	True (SG2-14)
DDSW	113	NA	NA	SG2-14	Lp	+	–	Growth	No Growth	SG2-14	Lp	True (SG2-14)
DDSW	127	NA	NA	SG2-14	Lp	+	–	Growth	No Growth	SG2-14	Lp	True (SG2-14)
RW	1	<0.04	0.31	SG1	Lp	+	+	Growth	No Growth	SG1	Lp	True (SG1)
RW	8	<0.04	<0.3	SG1	Lp	+	+	Growth	No Growth	SG1	Lp	True (SG1)
RW	26	<0.04	<0.3	SG2-14	Lp	+	–	Growth	No Growth	SG2-14	Lp	True (SG2-14)
RW	1	<0.04	<0.3	SG1	Lp	+	+	Growth	No Growth	SG1	Lp	True (SG1)
RW	4	<0.04	<0.3	SG1	Lp	+	+	Growth	No Growth	SG1	Lp	True (SG1)
DDSW	2	1.49	<0.3	No RXN	Lp	+	–	Growth	No Growth	No RXN	Lp	True (SG2-14)

Well Type	Initial Water Quality Results			Confirmation Results								Legiolert Determination
				Direct from Legiolert						From BCYE (+*cys*)		
	Lp Conc. (MPN/100 mL)	Free Chlorine (mg/L Cl_2)	Turbidity (NTU)	LA	MALDI-MS	qPCR (*mip*)	qPCR (*srkA*)	BCYE (+*cys*)	BCYE (-*cys*)	LA	MALDI-MS	
DDSW	1	0.99	<0.3	SG2-14	Lp	+	–	Growth	No Growth	SG2-14	Lp	True (SG2-14)
RW	5	6.20	<0.3	SG1	Lp	+	+	Growth	No Growth	SG1	Lp	True (SG1)
RW	1	<0.04	<0.3	SG1	Lp	+	+	Growth	No Growth	SG1	Lp	True (SG1)
RW	7	<0.05	<0.3	SG2-14	Lp	+	–	Growth	No Growth	SG2-14	Lp	True (SG2-14)
RW	1	<0.06	<0.3	SG2-14	Lp	+	–	Growth	No Growth	SG2-14	Lp	True (SG2-14)
RW	7	<0.07	<0.3	SG2-14	Lp	+	–	Growth	No Growth	SG2-14	Lp	True (SG2-14)
NA	NA	NA	NA	SG1	Lp	+	+	Growth	No Growth	SG1	Lp	True (SG1)
DDSW	2	1.22	1.30	SG2-14	Lp	+	+	Growth	No Growth	SG1	Lp	True (SG1)
RW	1	<0.04	<0.3	SG2-14	Lp	+	–	Growth	No Growth	SG2-14	Lp	True (SG2-14)
DDSW	2	0.06	0.33	SG1	Lp	+	+	Growth	No Growth	SG1	Lp	True (SG1)
RW	5	<0.04	<0.3	SG2-14	Lp	+	–	Growth	No Growth	SG2-14	Lp	True (SG2-14)
RW	1	<0.04	<0.3	SG2-14	Lp	+	–	Growth	No Growth	SG2-14	Lp	True (SG2-14)
DDSW	92	NA	NA	SG2-14	Lp	+	–	Growth	No Growth	SG2-14	Lp	True (SG2-14)
RW	1	<0.04	<0.3	SG2-14	Lp	+	–	Growth	No Growth	SG2-14	Lp	True (SG2-14)
DDSW	157	NA	NA	SG2-14	Lp	+	–	Growth	No Growth	SG2-14	Lp	True (SG2-14)
RW	7	<0.04	<0.3	SG1	Lp	+	+	Growth	No Growth	SG1	Lp	True (SG1)
RW	1	<0.04	<0.3	SG1	Lp	+	+	Growth	No Growth	SG2-14[e]	Lp	True (SG1&2-14)
RW	1	<0.04	<0.3	SG1	Lp	+	+	Growth	No Growth	SG1	Lp	True (SG1)
RW	8	<0.04	<0.3	SG2-14	Lp	+	–	Growth	No Growth	SG2-14	Lp	True (SG2-14)
DDSW	10	<0.04	<0.3	SG2-14	Lp	+	–	Growth	No Growth	SG2-14	Lp	True (SG2-14)
DDSW	15	0.40	0.48	No RXN	Lp	+	–	Growth	No Growth	No RXN	Lp	True (SG2-14)
DDSW	4	<0.04	26.3	No RXN	Lp	+	–	Growth	No Growth	No RXN	Lp	True (SG2-14)
DDSW	3	0.08	0.55	SG1	Lp	+	+	Growth	No Growth	SG1	Lp	True (SG1)
DDSW	5	1.39	0.34	SG1	Lp	+	+	Growth	No Growth	SG1	Lp	True (SG1)
DDSW	2	<0.04	1.08	SG1	Lp	+	+	Growth	No Growth	SG1	Lp	True (SG1)
DDSW	267	NA	NA	SG2-14	Lp	+	–	Growth	No Growth	SG2-14	Lp	True (SG2-14)

Well Type	Initial Water Quality Results			Confirmation Results								Legiolert Determination
				Direct from Legiolert						From BCYE (+*cys*)		
	Lp Conc. (MPN/100 mL)	Free Chlorine (mg/L Cl_2)	Turbidity (NTU)	LA	MALDI-MS	qPCR (*mip*)	qPCR (*srkA*)	BCYE (+*cys*)	BCYE (-*cys*)	LA	MALDI-MS	
DDSW	157	NA	NA	SG2-14	Lp	+	–	Growth	No Growth	SG2-14	Lp	True (SG2-14)
DDSW	1	0.21	<0.3	No RXN	Lp	+	–	Growth	No Growth	No RXN	Lp	True (SG2-14)
RW	4	5.80	<0.3	SG1	Lp	+	+	Growth	No Growth	SG1	Lp	True (SG1)
RW	4	<0.04	<0.3	SG1	Lp	+	+	Growth	No Growth	SG1	Lp	True (SG1)
RW	4	<0.04	<0.3	SG2-14	Lp	+	–	Growth	No Growth	SG2-14	Lp	True (SG2-14)
RW	19	<0.04	<0.3	SG2-14	Lp	+	–	Growth	No Growth	SG2-14	Lp	True (SG2-14)
RW	4	<0.04	<0.3	SG1	Lp	+	–	Growth	No Growth	SG1	Lp	True (SG1)
RW	1	<0.04	<0.3	No RXN	No ID	–	–	Growth	Growth	No RXN	No ID	False
RW	2	<0.04	<0.3	SG1	Lp	+	+	Growth	No Growth	SG1	Lp	True (SG1)
RW	26	<0.04	<0.3	SG1	Lp	+	+	Growth	No Growth	SG1	Lp	True (SG1)
RW	60	<0.04	<0.3	SG2-14	Lp	+	–	Growth	No Growth	SG2-14	Lp	True (SG2-14)
RW	3	<0.04	<0.3	SG1	NA[a]	+	+	Growth	No Growth	SG1	Lp	True (SG1)
RW	4	<0.04	<0.3	SG2-14	Lp	+	–	Growth	No Growth	SG2-14	Lp	True (SG2-14)
RW	9	<0.04	<0.3	SG2-14	Lp	+	–	Growth	No Growth	SG2-14	Lp	True (SG2-14)
RW	1	<0.04	<0.3	SG2-14	Lp	+	–	Growth	No Growth	SG2-14	Lp	True (SG2-14)
	**Confirmed Legiolert Result**			95	99	104	40	95		91	101	
				**88%**	**92%**	**96%**	**37%**	**88%**		**84%**	**94%**	
	**Contradicted Legiolert Result (or unable to confirm)**			13	9	4	68	13		17	7	
				**12%**	**8%**	**4%**	**63%**	**12%**		**16%**	**6%**	
	**Correct Determination of Sample**			93	100	106	105	97		93	102	106
				**86%**	**93%**	**98%**	**97%**	**90%**		**86%**	**94%**	**98%**
	**Incorrect Determination of Sample (or unable to make determination)**			15	8	2	3	11		15	6	2
				**14%**	**7%**	**2%**	**3%**	**10%**		**14%**	**6%**	**2%**

[a]Insufficient volume for test; [b]Not *Legionella pneumophila*; identified as *Stenotrophomonas maltophilia*; [c]Not *Legionella pneumophila*; identified as *Nocardia cyriacigeorgica*; [d]Simultaneous growth of other bacteria in Legiolert media likely confounded some confirmation testing; [e]Failed to identify SG1 in same sample but not necessarily incorrect

Table S2. qPCR assay details.

**Target**	**Primer** (5' → 3')	**Cycling Condition**	**Reference**
mip	F: TTGTCTTATAGCATTGGTGCCG<br>R: CCAATTGAGCGCCACTCATAG<br>P: CY5-CGGAAGCAATGGCTAAAGGCATGCA-BHQ1	1 cycle: 5 min at 98°C,<br>35 cycles: 95°C for 120s, 58°C for 10s, and 72°C for 30s<br>For melt curve (*srkA* only): 65°C to 95°C in 0.5°C increments, continuous fluorescence acquisition	Toplitsch et al. 2018
srkA	F: AAACGCCTCTTTGCTGAACCAG<br>R: GTTGGGCATCTTCTTGATTAATCC		Nakaue et al. 2021

Figure S1. Summary of log_{10}-transformed *L. pneumophila* concentrations for Legiolert-positive samples. The box-and-whisker plot is presented in the Tukey style, with boxes indicating the 25th, 50th (median), and 75th percentiles; whiskers denoting ±1.5 times the interquartile range (IQR); circles representing outliers based on the IQR method; and the x symbol indicating the log_{10}-transformed mean.

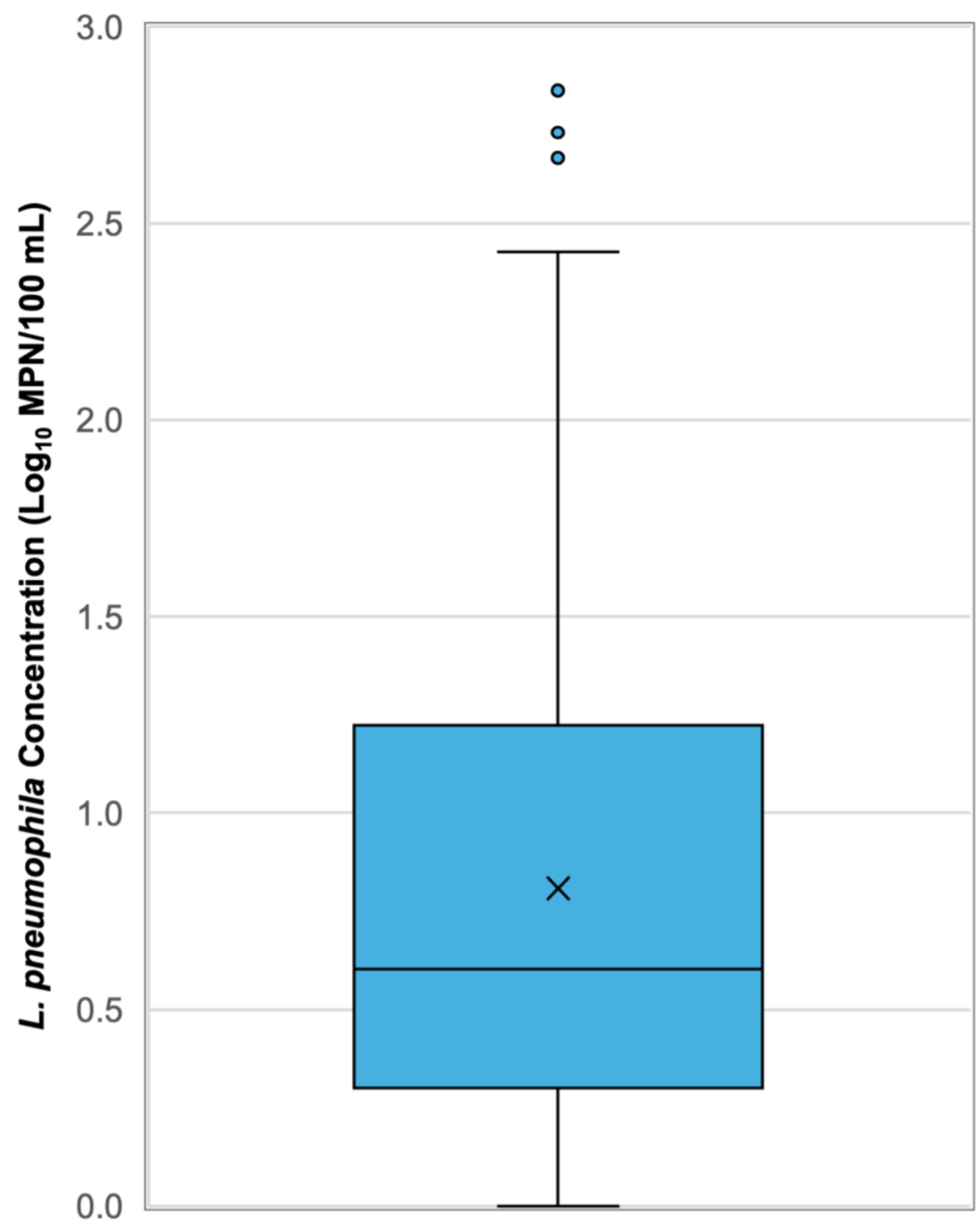

Supplement: Supplementary file 1 [file spectrum.01228-26-s0001.pdf]
